# Supplementary material for: Single-cell chromatin accessibility landscape in kidney identifies additional cell-of-origin in heterogenous papillary renal cell carcinoma
Source: Nat Commun. 2022 Jan 10;13:31. doi: 10.1038/s41467-021-27660-3 (PMC8748507; doi:10.1038/s41467-021-27660-3)
Supplement: Supplementary file 1 — Supplementary Information [file 41467_2021_27660_MOESM1_ESM.pdf]

Supplementary Information

Single-cell chromatin accessibility landscape in kidney identifies additional cell-of-origin in heterogenous papillary renal cell carcinoma

Qi Wang[1 #], Yang Zhang[1,2 #], Bolei Zhang[3], Yao Fu[4], Xiaozhi Zhao[5], Jing Zhang[1], Ke Zuo[2], Yuexian Xing[2], Song Jiang[2], Zhaohui Qin[6], Erguang Li[1], Hongqian Guo[5,*], Zhihong Liu[1,2,*], Jingping Yang[1,2,7*]

[1] Medical School of Nanjing University, Nanjing, Jiangsu, 210093, China;

[2] National Clinical Research Center for Kidney Disease, Jinling Hospital, Medical School of Nanjing University, Nanjing, Jiangsu, 210002, China;

[3] School of computer science, Nanjing university of posts and telecommunications, Nanjing, Jiangsu, 210023, China;

[4] Department of Pathology, affiliated Drum Tower Hospital, Medical School of Nanjing University, Nanjing, Jiangsu, 210008, China;

[5] Department of Urology, affiliated Drum Tower Hospital, Medical School of Nanjing University, Nanjing, Jiangsu, 210008, China;

[6] Department of Biostatistics and Bioinformatics, Rollins School of Public Health, Emory University, Atlanta, GA 30322, USA;

[7]Jiangsu Key Laboratory of Molecular Medicine, Medical School, Nanjing University, Nanjing 210093, China;

# These authors contributed equally: Q.W. and Y.Z.

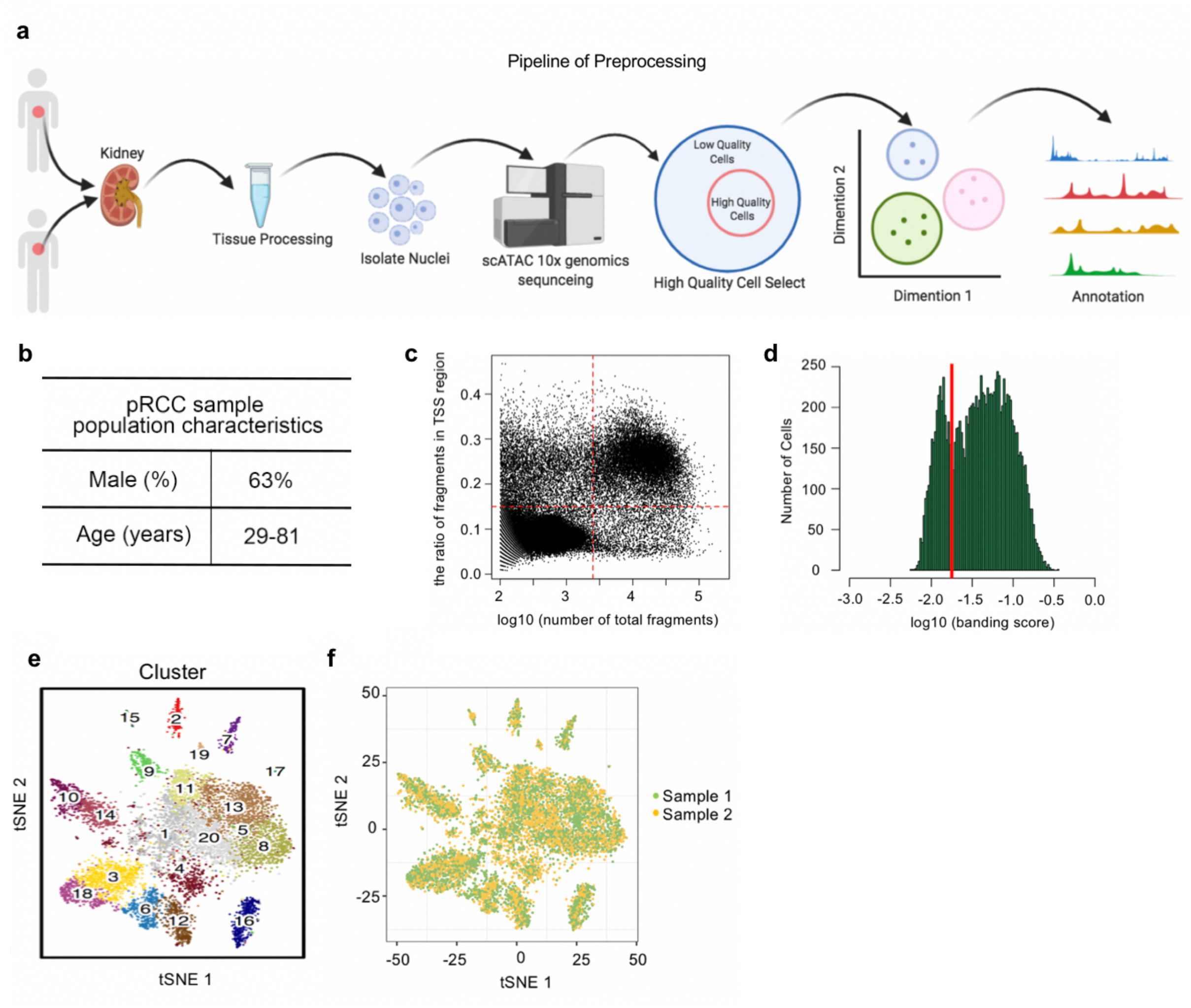

Supplementary Figure 1 | Preprocessing of human kidney scATAC-seq data. a, Pipeline of preprocessing human kidney scATAC-seq data. **b,** Characteristics of all pRCC samples in this study. **c,** Quality control based on distribution of log10 (number of total fragments) and the ratio of fragments in TSS region per cell. Each dot represents a single cell, the dotted lines indicate log10 (number of total fragments) of 3.4 and the ratio of fragments in TSS region of 0.15. **d,** Quality control based on banding score distribution per cell. The red line indicates a log10 (banding score) of -1.75. **e,** t-SNE embedding of cells colored by cluster. **f,** t-SNE embedding of cells colored by sample ID.

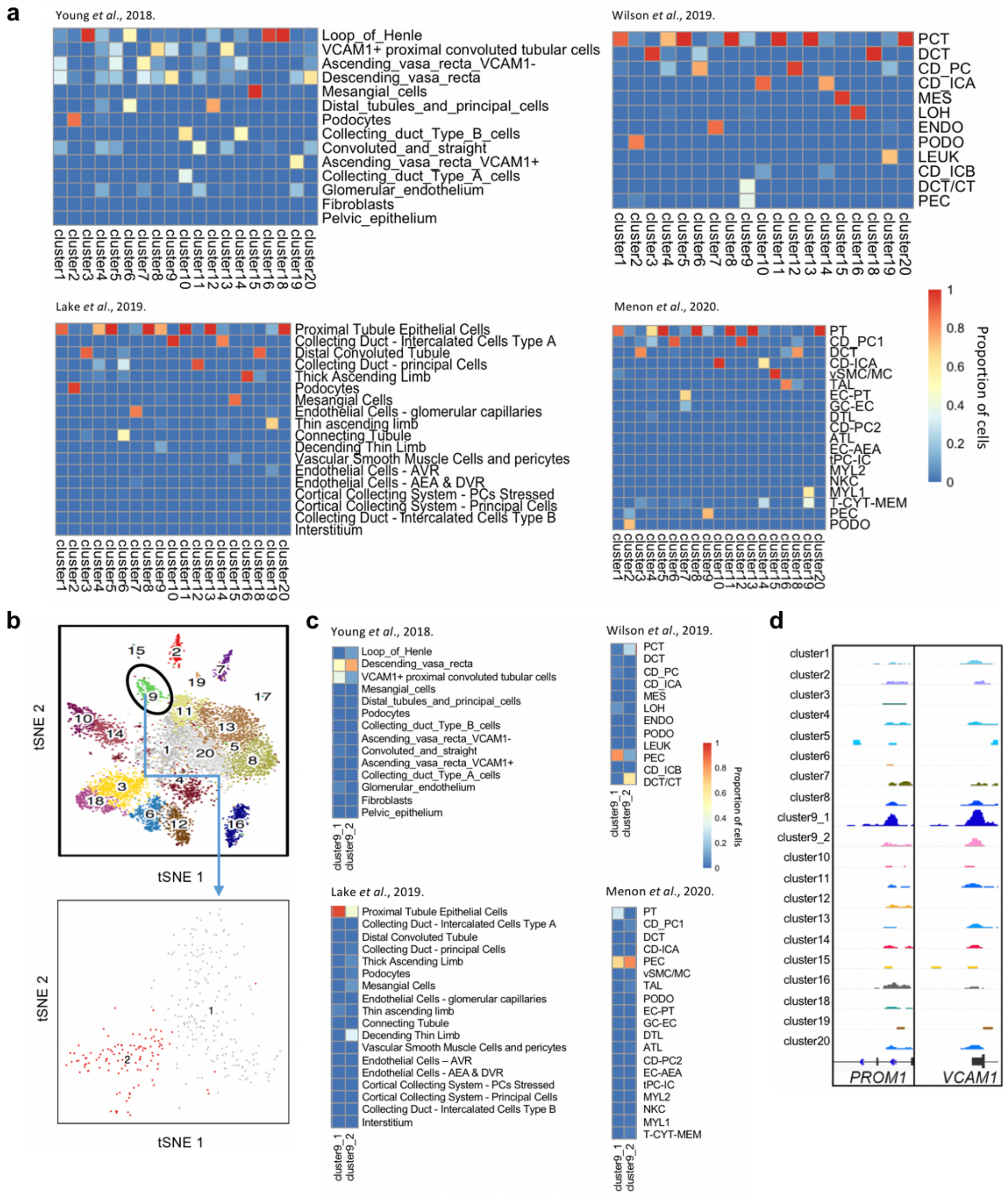

Supplementary Figure 2 | Integrating scATAC-seq with four scRNA-seq datasets. a, Matrix of the proportions of cells in each scATAC-seq cluster that transferred with cell labels from scRNA-seq datasets. **b,** Re-cluster of cluster9. Grey dots represent cluster9_1 and red dots represent cluster9_2. **c,** Matrix of the proportions of cells in cluster9_1 and cluster9_2 that

transferred with cell labels from scRNA-seq datasets. **d,** Genome browser view of aggregated chromatin accessibility at *VCAM1* and *PROM1* gene loci for each cluster.

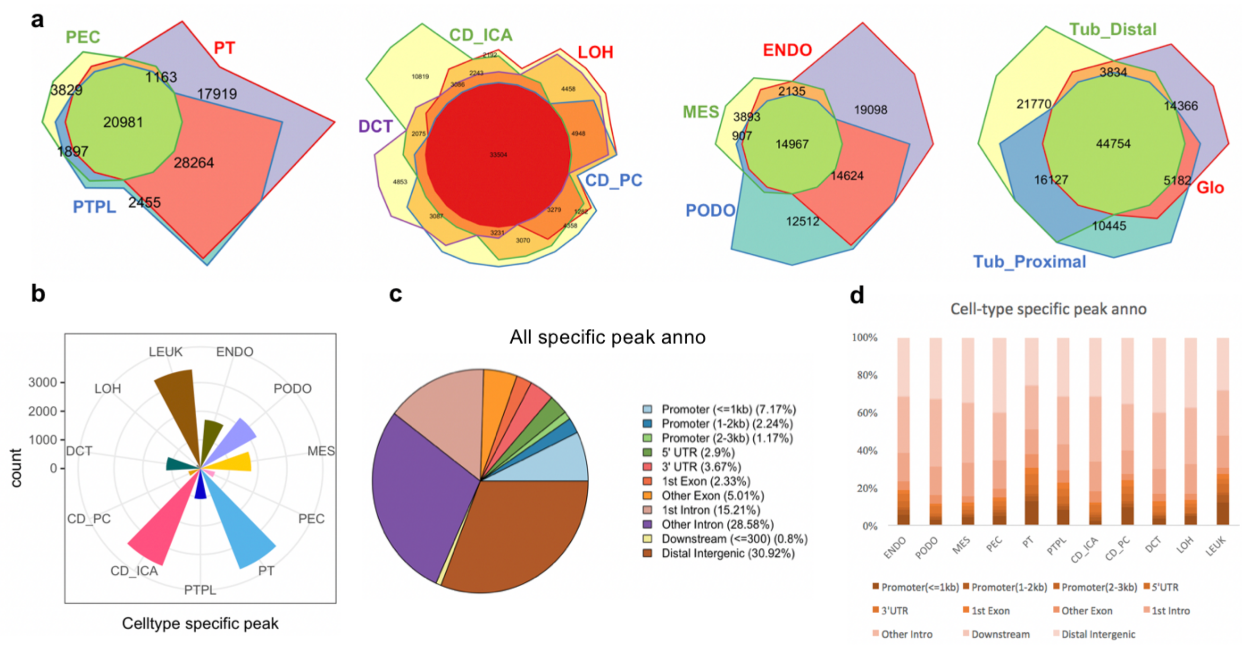

Supplementary Figure 3 | Comparison of ATAC peaks among the cell types. a, Chow-Ruskey plots of ATAC clean peaks between cell types within proximal tubule, between cell types within distal tubules, between cell types within glomerulus, or between these kidney compartments as proximal tubule, distal tubule and glomerulus. Clean peaks in each cell type were mapped to the master peak list using “findOverlaps” function in GenomicRanges. **b,** Number of specific peaks in each cell type. **c,** Genomic distribution for overall cell type-specific peaks. **d,** Genomic distribution for cell type-specific peaks in each cell type.

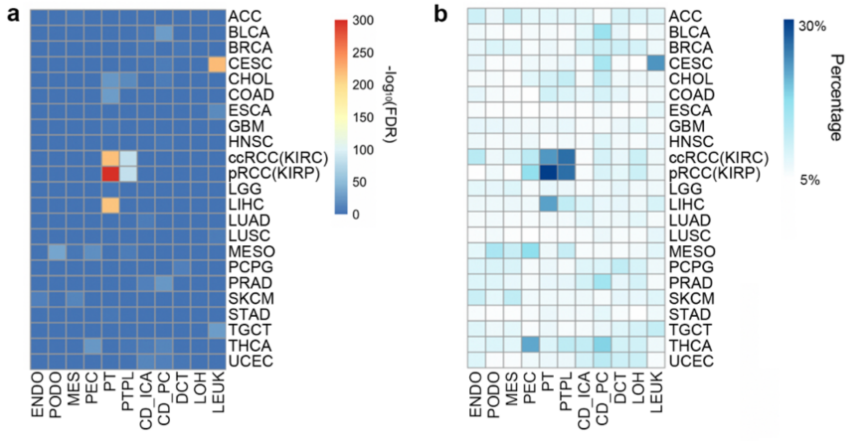

Supplementary Figure 4 | Overlap between kidney cell type-specific peaks and cancer type-specific peaks. a, Heatmap showing significance of the overlap between kidney cell type-specific peaks and cancer type-specific peaks. Colors indicate the –log10(FDR) of the overlapping. **b**, Heatmap showing the overlap between kidney cell type-specific peaks and cancer type-specific peaks. Colors indicate the percentage of kidney cell type-specific peaks that overlap with cancer peaks.

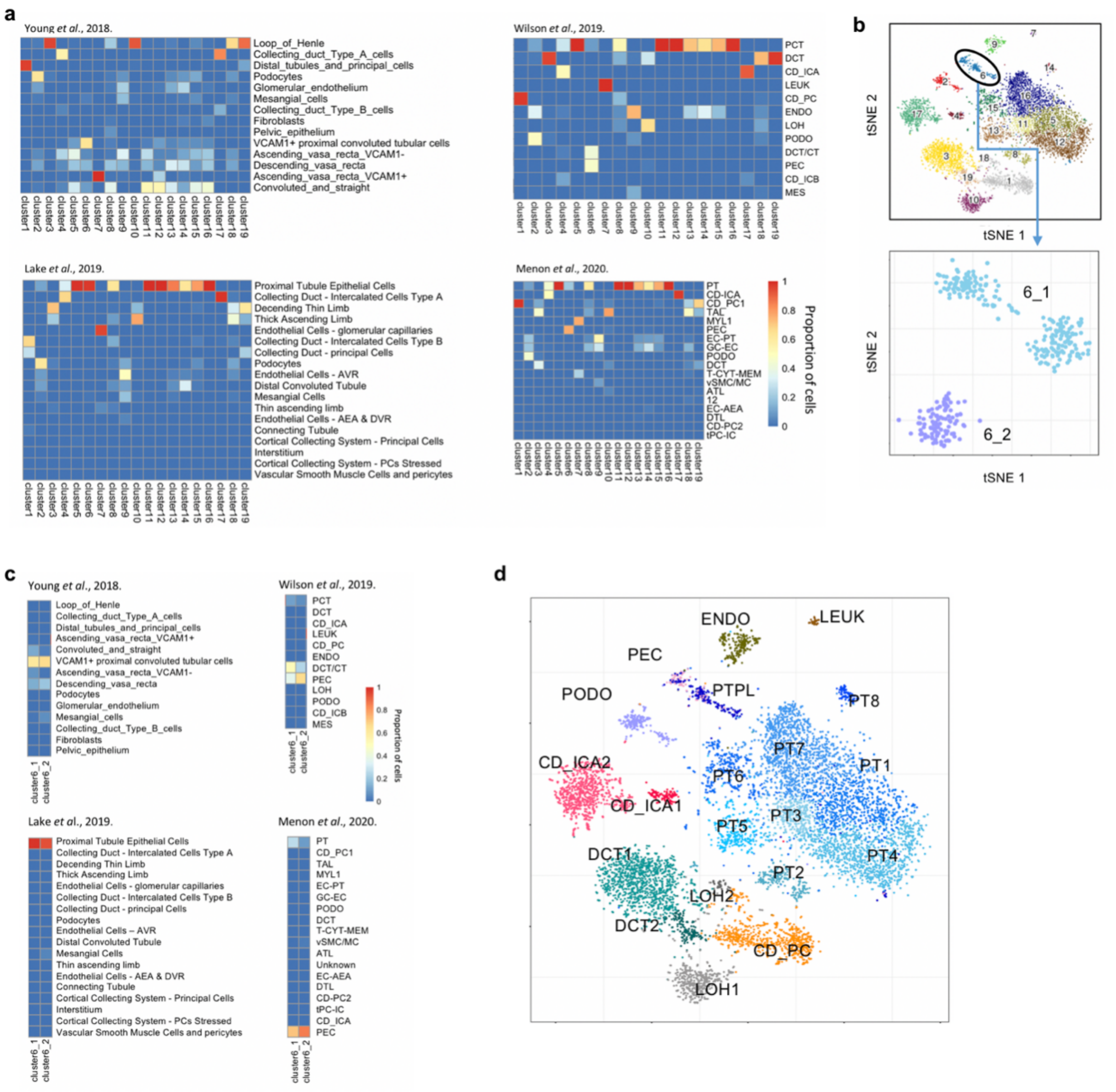

Supplementary Figure 5 | scATAC-seq clusters from cisTopic. a, Annotation matrix of scATAC-seq clusters from cisTopic. **b**, Re-cluster of cluster6 which is not well annotated. Light blue dots represent cluster6_1 and light purple dots represent cluster6_2. **c**, Matrix of the proportions of cells in cluster6_1 and cluster6_2 that transferred with cell labels from scRNA-seq datasets. **d**, t-SNE embedding of all cells colored by cisTopic clustering.

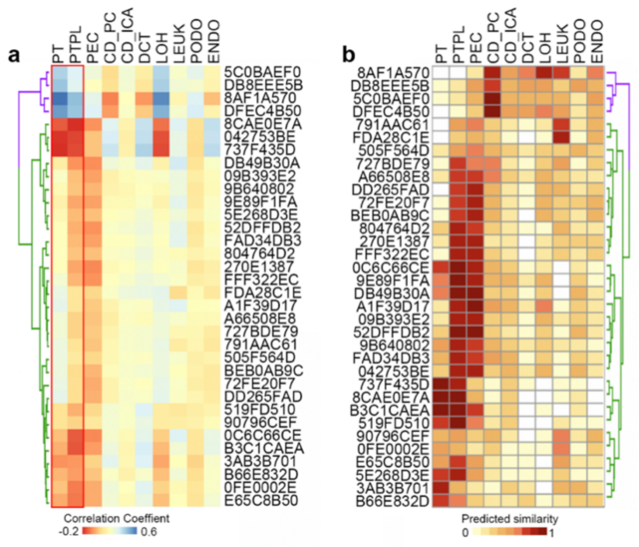

Supplementary Figure 6 | Comparison of normal kidney cell types clustered by cisTopic and cancer ATAC-seq. a-b, Pearson correlations (a) or similarity scores (b) between normalized chromatin accessibilities in 34 pRCC samples and normal kidney cell types analyzed with cisTopic. Hierarchical clustering of pRCC samples based on the correlations or similarity scores revealed two subgroups, and heatmap view of the correlations or similarity scores show two different accessibility correlation patterns in the 34 pRCC samples. Each row represents a pRCC sample and each column represents a cell type. The red box highlighted the PT cell type groups. The patients' prefixes of Stanford UUIDs are indicted for each sample. Source data are provided as a Source Data file.

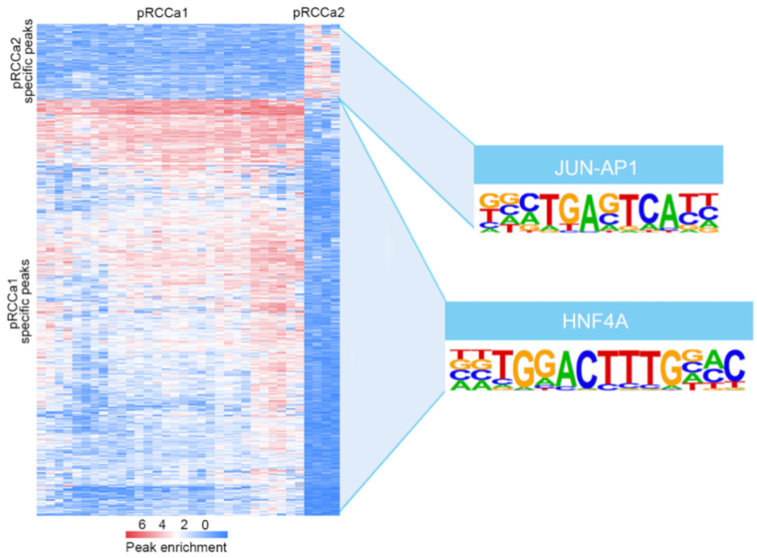

Supplementary Figure 7 | pRCCa1 and pRCCa2 differential peaks. Heatmap of differential peaks between pRCC subgroups (left). Each row represents a differential peak, and each column represents a pRCC sample. Top enriched motif in pRCCa1- or pRCCa2- specific peaks (right).

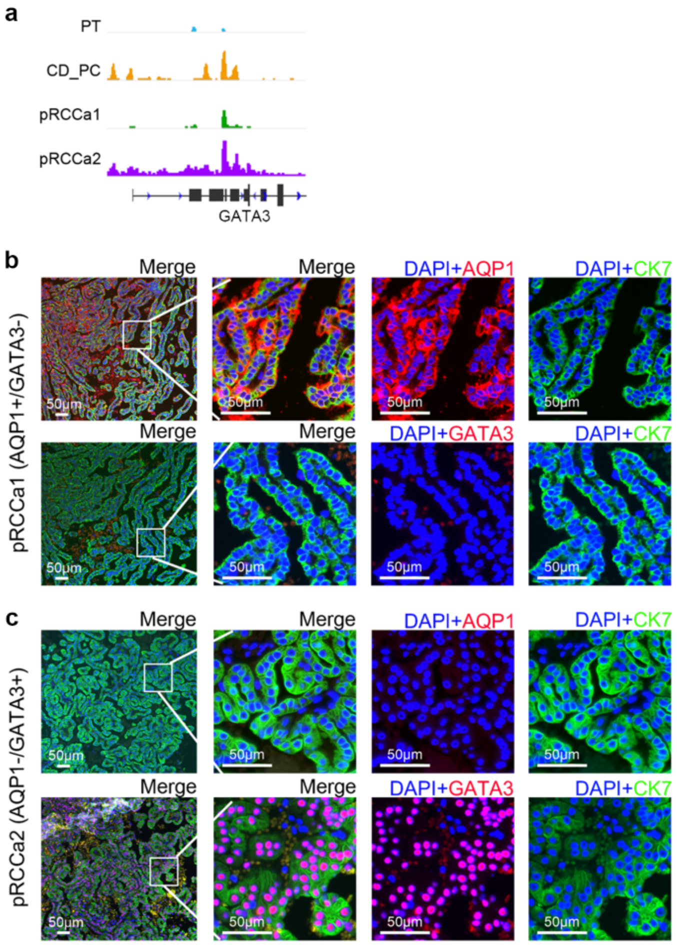

Supplementary Figure 8 | GATA3 in pRCC. a, Genome browser view in PT, CD_PC, pRCCa1 and pRCCa2 at CD_PC marker, *GATA3* locus. **b-c,** Immunofluorescence staining of tumor tissues with AQP1 (red), GATA3 (red), pRCC marker cytokeratin 7 (CK7, green) and DAPI (blue). A group of patients show colocalization of AQP1 and CK7 and absence of GATA3 (b). Data is from one experiment representative of 48 independent experiments samples examined. A second group of patients show localization of GATA3 in the cell nuclei with CK7 but absence of AQP1 (c). Data is from one experiment representative of 2 independent samples examined.

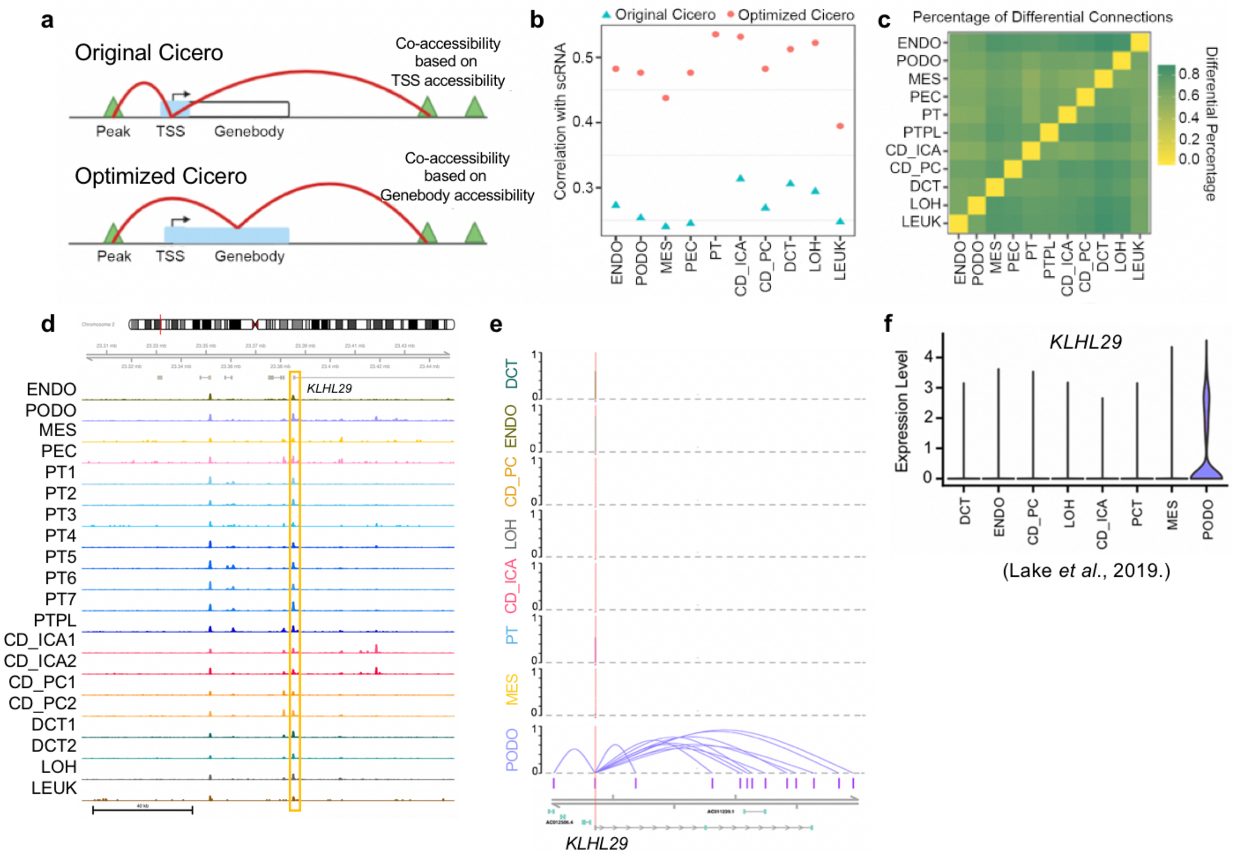

Supplementary Figure 9 | Cell type-specific regulatory networks established with optimized Cicero. a, Strategy of optimized Cicero network construction. **b,** Pearson correlation between expression from scRNA-seq datasets and gene activity scores calculated from regulatory networks. **c,** Comparison of peaks connection in regulatory networks between cell types. Color represents the percentage of differential connections between cell types. **d,** Genome browser view of aggregated chromatin accessibility at *KLHL29* loci which is differentially regulated. The yellow box highlights *KLHL29* promoter. **e,** Connections to *KLHL29* promoter in regulatory networks. Arcs show peaks connection, and the heights of arcs indicate strength of the connections. Only connections with co-accessibility above 0.5 are shown. Viewpoint: *KLHL29* promoter. The purple bars in bottom track indicate scATAC-seq peaks. **f,** Violin plot of cell type-specific expression of *KLHL29* from scRNA-seq.

a

Origin-derived features	
pRCCa1	pRCCa2
AC004691.2	*B4GALNT3*
AC012651.1	*EHF*
ALPL	*GATA3*
ANPEP	*GRHL2*
AQP1	*HK1*
CAPN3	*MECOM*
CSDC2	*TFAP2A*
DPEP1	
ENPEP	
FRMD1	
GDA	
GLYAT	
GREB1	
HNF4A	
IGFBP4	
IQSEC3	
KBTBD11	
MME	
PCK1	
SLC12A7	
SLC47A1	
SLC6A13	
SLC6A19	
TMEM200A	
ZEB2	

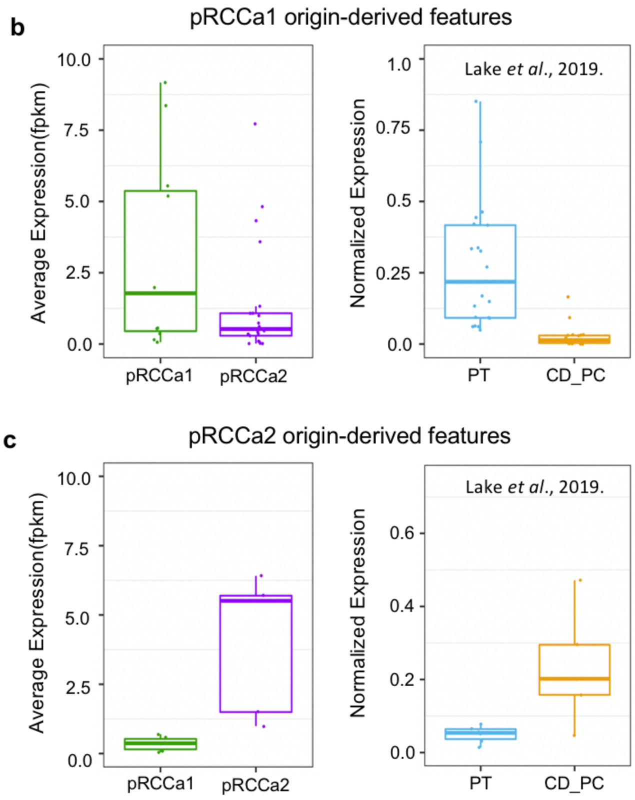

Supplementary Figure 10 | Expression of origin-derived feature genes. a, Origin-derived features for pRCCa1 and pRCCa2. **b-c,** Expression of pRCCa1 (b) or pRCCa2 (c) origin-derived features in pRCCa1, pRCCa2, PT, CD_PC. Expressions of pRCCa1 and pRCCa2 are from bulk RNA-seq in TCGA. Expressions of PT and CD_PC cells are from scRNA-seq. The scRNA-seq was normalized by LogNormalize method. Box plots depict the median, quartiles and range. n=25 genes for pRCCa1 origin-derived features and n=7 genes for pRCCa2 origin-derived features. Source data are provided as a Source Data file.

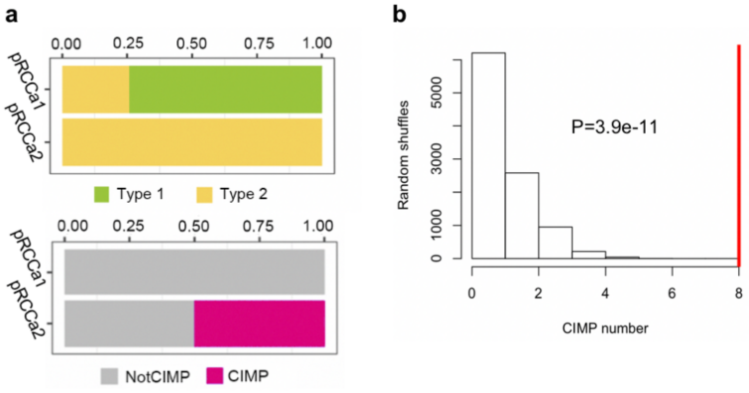

Supplementary Figure 11 | Pathological, molecular and clinical classifications of pRCC subgroups with different cell-of-origin. a, Percentage of pathological Type 1 and Type 2 (top) or CIMP (bottom) in pRCCa1 or pRCCa2. **b,** Distribution of numbers of CIMP in 41 randomly picked samples. The random picking of 41 out of 255 samples was repeated 10000 times. The red line denotes the number 8 which is the number of CIMP in the 41 samples as pRCCa2_Like. "pnorm" function was used to calculate the probability of number 8 with the following parameters: 'lower.tail=FALSE'. Two-sided t-test was used.

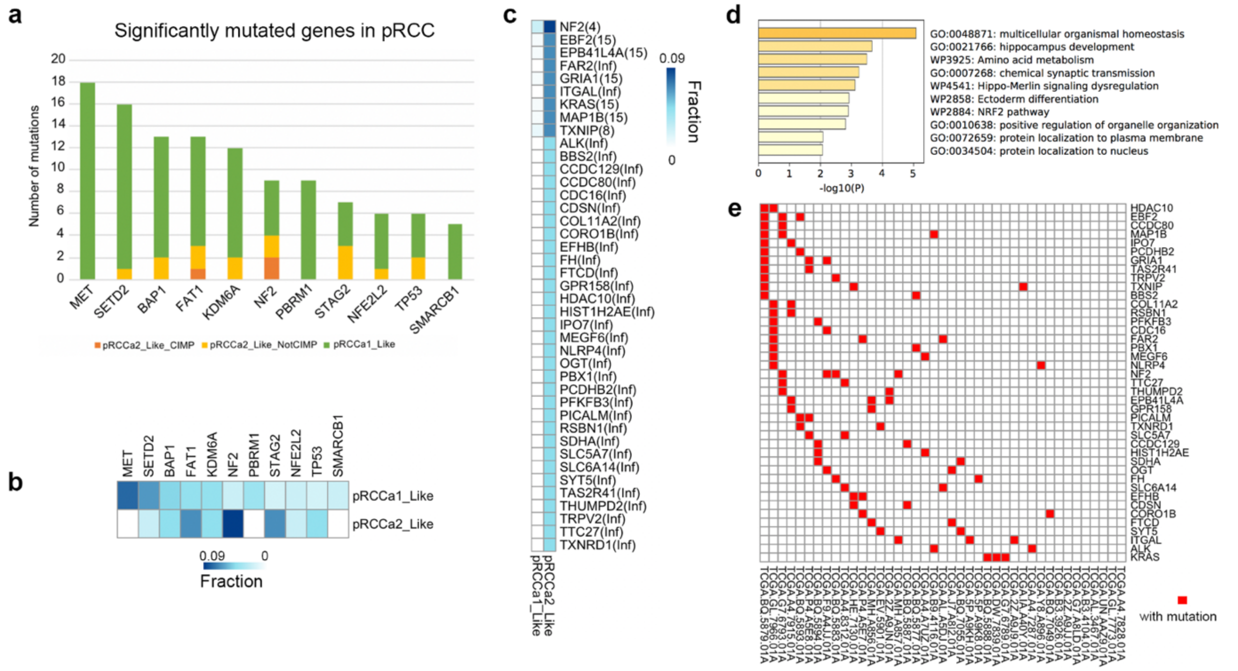

Supplementary Figure 12 | Somatic mutations in pRCC subgroups with different cell-of-origin. a, Distribution of mutations of 11 pRCC Significantly mutated genes (SMGs) [1] in the subgroups. **b**, Fraction of patients with mutation for each SMG in pRCC subgroups shows mutations in NF2 are enriched in pRCCa2_Like. **c,** List of genes whose mutations are significantly enriched in pRCCa2_Like subgroup (right). Frequency of mutations are counted in each subgroup. Genes with frequency odds ratio>1 in pRCCa2_Like and Fisher's exact test p-value<0.05 are listed. The fraction of patients with mutations for each of the listed genes are presented for the pRCC subgroups (left). Odds ratios are shown in brackets, and Inf indicates the mutations are exclusive in pRCCa2_Like. **d**, Pathway enrichment result from Metascape of pRCCa2_Like enriched mutation genes. **e**, Distribution of mutations of pRCCa2_Like enriched mutation genes in individual pRCCA2_Like patients. The patients' TCGA IDs are presented.

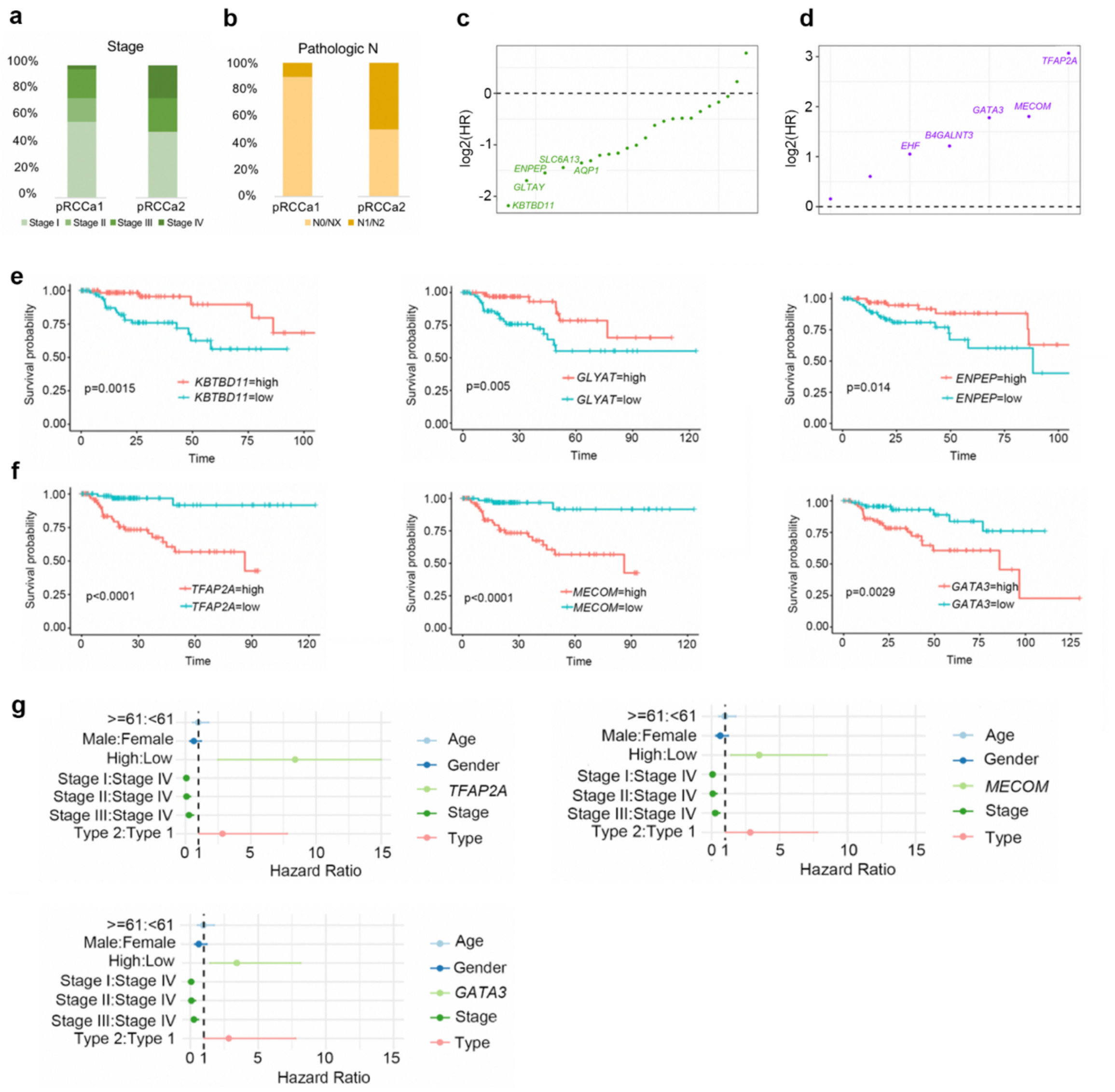

Supplementary Figure 13 | Origin-derived features predict survival in pRCC. a, Distribution of tumor stages in pRCCa1 or pRCCa2. **b**, Percentage of metastasis to regional lymph nodes in pRCCa1 or pRCCa2. N: regional lymph nodes. N0: no regional lymph node metastasis; NX: regional lymph nodes cannot be assessed; N1/N2: metastasis in regional lymph node(s). **c-d,** Hazard ratio from survival analysis for pRCCa1 (c) or pRCCa2 (d) origin-derived features. The dash line shows log2 (HR)=0 which indicated no risk association. **e-f,** Kaplan-

Meier analysis for top 3 pRCCa1 (e) or pRCCa2 (f) origin-derived features stratified by feature gene expression. Feature gene expression was divided into “high” and “low” representing the upper and lower quarter of gene expression. The Log-rank test was used to assess the univariate survival differences. **g,** Hazard ratio for top 3 pRCCa2 origin-derived features on pRCC survival. The Cox Proportional Hazard model was used to calculate hazard ratio. The binary values of feature gene expression, age, gender, stage, and tumor type are used as covariates. Lines represent 95% confidence intervals. The dash lines show HR=1 which indicated no risk association.

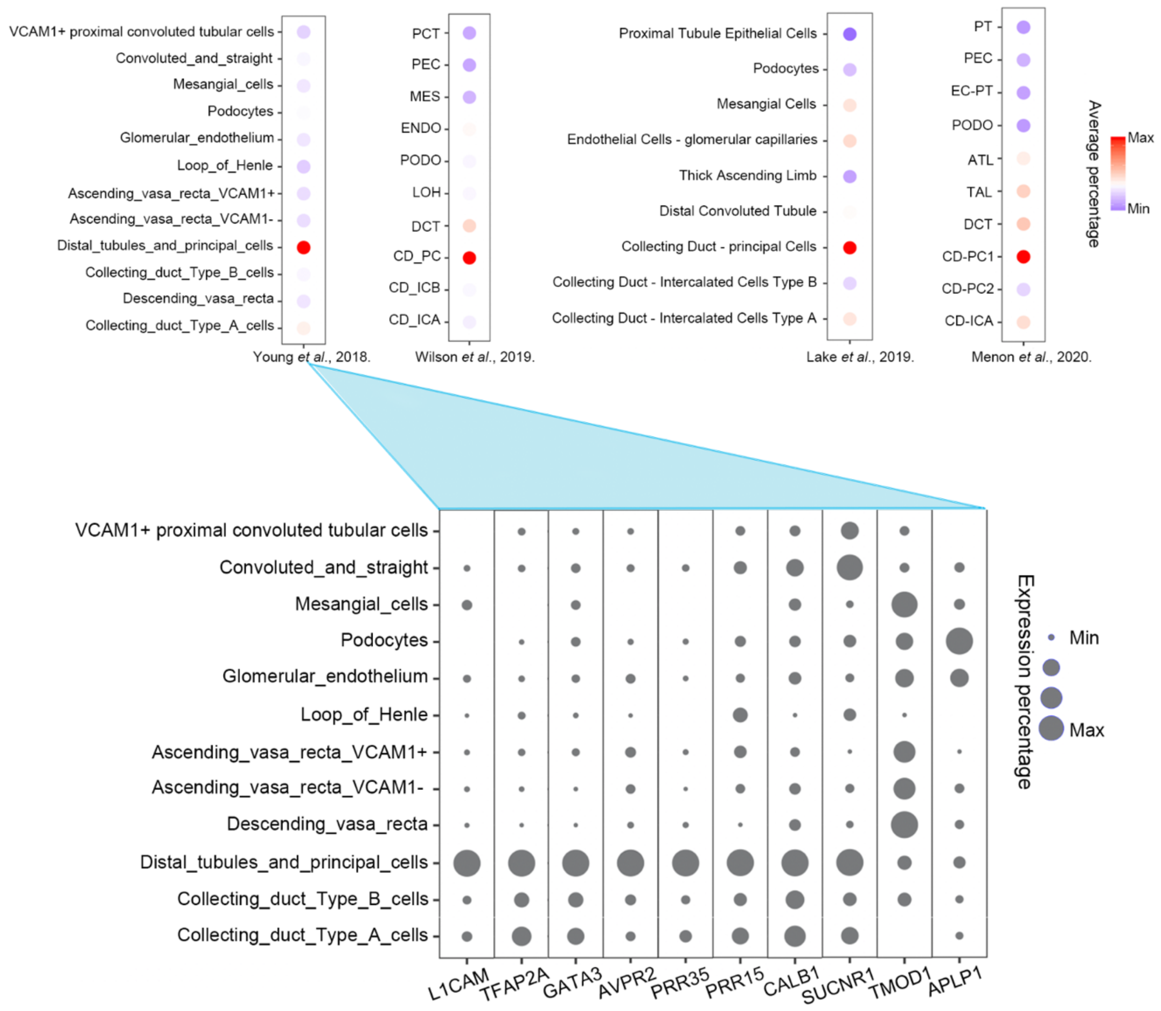

Supplementary Figure 14 | Expression pattern of CIMP associated genes. Expression of identified top gene features for CIMP in four scRNA-seq datasets (top). Top 10 gene features with expression in more than 10% cells in at least one cell type were measured. The percentage of cells with expression was scaled between cell types. The average of all genes in each cell type was then taken as the expression level of the CIMP associated gene panel in that cell type. The detailed expression for each gene is plotted for the first scRNA-seq dataset as an example (bottom).

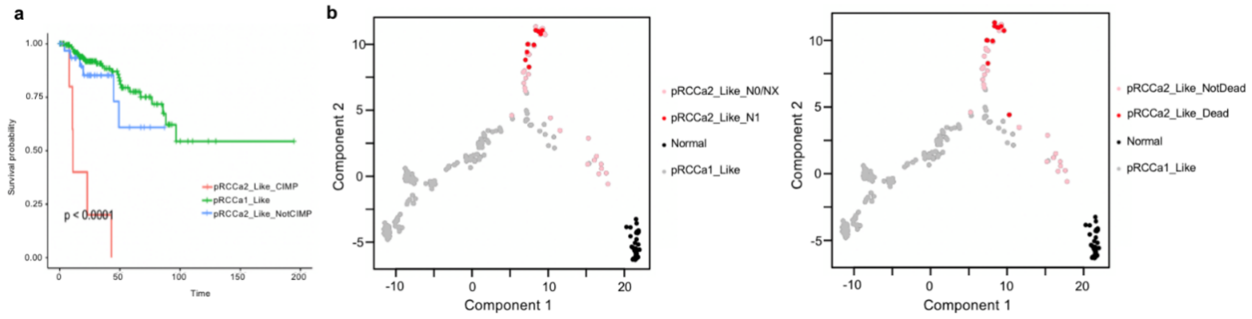

Supplementary Figure 15 | Clinical performance along pRCCa2_Like branch. a, Survival analysis for pRCCa1_Like, pRCCa2_Like_CIMP and pRCCa2_Like_NotCIMP. **b,** Trajectory the same as Fig. 3e, but colored by state of metastasis to regional lymph nodes (left) or death (right) along the trajectory in pRCCa2_Like branch. N0: No regional lymph node metastasis; NX: Regional lymph nodes cannot be assessed; N1: Metastasis in regional lymph node(s).

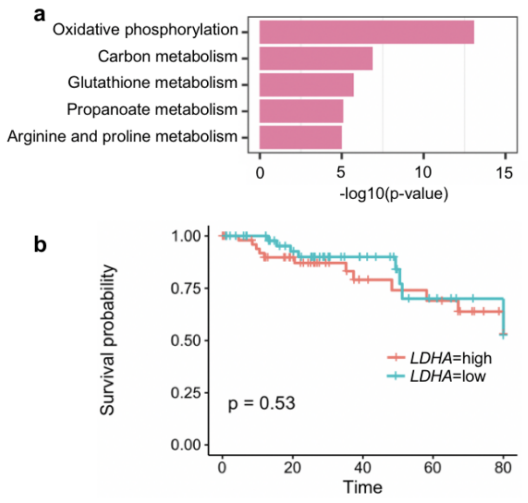

Supplementary Figure 16 | Metabolic reprogramming in pRCC. a, Top 5 enriched KEGG terms for differential genes between pRCCa2_Like_CIMP and pRCCa2_Like_NotCIMP highlight metabolic reprogramming in advanced pRCCa2_Like subgroup. **b,** Kaplan-Meier analysis of overall survival stratified by *LDHA* expression in pRCCa1_Like subgroup. *LDHA* gene expression was divided into "high" and "low" representing the upper and lower quarter of *LDHA* gene expression. The Log-rank test was used to assess the univariate survival differences. Source data are provided as a Source Data file.

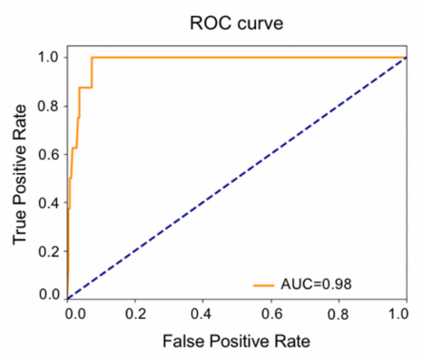

Supplementary Figure 17 | Prediction for CIMP. ROC curve of predicting CIMP based on Random Forest model with the origin-derived features and LDHA.

Supplementary References

1. Lawrence MS*, et al.* Discovery and saturation analysis of cancer genes across 21 tumour types. *Nature* **505**, 495-+ (2014).
