# Supplementary material for: Single-cell chromatin accessibility landscape in kidney identifies additional cell-of-origin in heterogenous papillary renal cell carcinoma
Source: Nat Commun. 2022 Jan 10;13:31. doi: 10.1038/s41467-021-27660-3 (PMC8748507; doi:10.1038/s41467-021-27660-3)
Supplement: Supplementary file 3 — Description of Additional Supplementary Files [file 41467_2021_27660_MOESM3_ESM.pdf]

Description of Additional Supplementary Files

File Name: Supplementary Data 1
Description: Celltype and batch information for each barcode.

File Name: Supplementary Data 2
Description: **All mutations in 255 TCGA pRCC samples.**
