# Supplementary material for: Single-cell chromatin accessibility landscape in kidney identifies additional cell-of-origin in heterogenous papillary renal cell carcinoma
Source: Nat Commun. 2022 Jan 10;13:31. doi: 10.1038/s41467-021-27660-3 (PMC8748507; doi:10.1038/s41467-021-27660-3)
Supplement: Supplementary file 6 — Reporting Summary [file 41467_2021_27660_MOESM6_ESM.pdf]

nature research

Corresponding author(s): Jingping Yang
Last updated by author(s): Oct 1, 2021

Reporting Summary

Nature Research wishes to improve the reproducibility of the work that we publish. This form provides structure for consistency and transparency in reporting. For further information on Nature Research policies, see our Editorial Policies and the Editorial Policy Checklist.

Statistics

For all statistical analyses, confirm that the following items are present in the figure legend, table legend, main text, or Methods section.

n/a	Confirmed	
☐	☒	The exact sample size (*n*) for each experimental group/condition, given as a discrete number and unit of measurement
☐	☒	A statement on whether measurements were taken from distinct samples or whether the same sample was measured repeatedly
☐	☒	The statistical test(s) used AND whether they are one- or two-sided<br>*Only common tests should be described solely by name; describe more complex techniques in the Methods section.*
☒	☐	A description of all covariates tested
☐	☒	A description of any assumptions or corrections, such as tests of normality and adjustment for multiple comparisons
☐	☒	A full description of the statistical parameters including central tendency (e.g. means) or other basic estimates (e.g. regression coefficient) AND variation (e.g. standard deviation) or associated estimates of uncertainty (e.g. confidence intervals)
☐	☒	For null hypothesis testing, the test statistic (e.g. *F*, *t*, *r*) with confidence intervals, effect sizes, degrees of freedom and *P* value noted<br>*Give P values as exact values whenever suitable.*
☒	☐	For Bayesian analysis, information on the choice of priors and Markov chain Monte Carlo settings
☒	☐	For hierarchical and complex designs, identification of the appropriate level for tests and full reporting of outcomes
☒	☐	Estimates of effect sizes (e.g. Cohen's *d*, Pearson's *r*), indicating how they were calculated

Our web collection on statistics for biologists contains articles on many of the points above.

Software and code

Policy information about availability of computer code

Data collection	No software was used for data collection.
Data analysis	Adapter sequences were trimmed using trimmomatic (0.32). Bowtie2 (2.3.0) was used to align reads to the hg38 reference genome and then filtered out using samtools (1.3.1). SnapATAC (1.0.0) were used for clustering analysis and Seurat(3.0.0) was used for annotating cluster identity. MACS2 (2.1.1) was used for peak calling and merged by bedtools (2.25.0). Motif analysis was performed by HOMER (4.11.1) and chromVAR (1.4.1). Comparison by similarity score between normal kidney cell types and 34 pRCC samples was performed by R package glmnet (4.1-1). Differential analysis between pRCC subtypes was performed using edgeR (3.24.3). Regulatory networks were constructed by R package Cicero (1.0.15). R (3.5.1), ChIPseeker (1.18.0), GenomicRanges (1.34.0), Cluster3, Treeview (1.1.6r4), IGV (2.3.92), bedGraphToBigWig (v4), GREAT (4.0.4), GSEA (4.1.0), DisGeNET (in Enrichr 2016), Enrichr (2016 updated), Metascape (version2019), Monocle (version 2), scikit-learn (V.0.4.2) and Python(2.7.10).

For manuscripts utilizing custom algorithms or software that are central to the research but not yet described in published literature, software must be made available to editors and reviewers. We strongly encourage code deposition in a community repository (e.g. GitHub). See the Nature Research guidelines for submitting code & software for further information.

Data

Policy information about availability of data

All manuscripts must include a data availability statement. This statement should provide the following information, where applicable:
- Accession codes, unique identifiers, or web links for publicly available datasets
- A list of figures that have associated raw data
- A description of any restrictions on data availability

The scRNA-seq publicly available data used in this study are available in the GEO database under accession code GSE140989, GSE121862, GSE131882 or

downloaded from https://science.sciencemag.org/highwire/filestream/713964/field_highwire_adjunct_files/6/aat1699_DataS1.gz.zip. The raw scATAC-seq data, master peak list and bigwig files generated in this study have been deposited in the GEO database under accession code GSE166547 and the raw scATAC-seq data is also available at the GSA for human database under accession code HRA001419. The remaining data supporting the findings of this study are available within the Article, Supplementary Information or Source Data file.

Field-specific reporting

Please select the one below that is the best fit for your research. If you are not sure, read the appropriate sections before making your selection.

- [x] Life sciences
- [ ] Behavioural & social sciences
- [ ] Ecological, evolutionary & environmental sciences

For a reference copy of the document with all sections, see nature.com/documents/nr-reporting-summary-flat.pdf

Life sciences study design

All studies must disclose on these points even when the disclosure is negative.

Sample size

No sample size calculation was performed for scATAC. According to publications, two biological replications are necessary for single cell analysis. As 4 out of 34 pRCC samples were found with alternative cell-of-origin through scATAC analysis, we choose sample size larger than 42 (34/4*5) for 5 fold chance to find one patient with alternative cell-of-origin in immunofluorescence staining experiments. In this study, we stained for 52 samples.

Data exclusions

No data were excluded from the analyses.

Replication

Two biological replications were performed independently for scATAC.

Randomization

Randomization of human participants was not relevant to our study, because we did not allocate samples into different groups.

Blinding

Blinding is not relevant to our study because we did not require separate experimental groups.

Reporting for specific materials, systems and methods

We require information from authors about some types of materials, experimental systems and methods used in many studies. Here, indicate whether each material, system or method listed is relevant to your study. If you are not sure if a list item applies to your research, read the appropriate section before selecting a response.

Materials & experimental systems

n/a	Involved in the study	
[ ]	[x]	Antibodies
[x]	[ ]	Eukaryotic cell lines
[x]	[ ]	Palaeontology and archaeology
[x]	[ ]	Animals and other organisms
[ ]	[x]	Human research participants
[x]	[ ]	Clinical data
[x]	[ ]	Dual use research of concern

Methods

n/a	Involved in the study	
[x]	[ ]	ChIP-seq
[x]	[ ]	Flow cytometry
[x]	[ ]	MRI-based neuroimaging

Antibodies

Antibodies used

DAPI (ab104139, Abcam, 1:1 diluted); mouse anti-AQP1 (ab9566; Abcam, 1:100 diluted); rabbit anti-GATA3 (ZA-0661; ZSGB-BIO, 1:1 diluted), rabbit anti-GRHL2 (HPA004820; Sigma-Aldrich, 1:50 diluted), rabbit anti-CK7 (ZA-0573; ZSGB-BIO, 1:1 diluted) and mouse anti-CK7 (ZM-0071; ZSGB-BIO, 1:1 diluted).

Validation

All antibodies are commercial available and were validated by the antibody manufacturer.

DAPI (ab104139, Abcam)
https://www.abcam.com/mounting-medium-with-dapi-aqueous-fluoroshield-ab104139.html
Validated for IHC in adult mouse by the manufacturer. This antibody has been cited in 327 publications.

Anti-AQP1 (ab9566; Abcam)
https://www.abcam.com/aquaporin-1-antibody-122-ab9566.html
Validated for IHC in rat brain by the manufacturer. This antibody has been cited in 52 publications.

Anti-GATA3 (ZA-0661; ZSGB-BIO)
http://www.zsbio.com/product/ZA-0661
Validated for IHC in human breast by the manufacturer.

Anti-GRHL2 (HPA004820; Sigma-Aldrich)
https://www.sigmaaldrich.cn/CN/zh/product/sigma/hpa004820?context=product
Validated for IHC in human kidney by Human Protein Atlas（HPA）.

Anti-CK7 (ZA-0573; ZSGB-BIO)
http://www.zsbio.com/product/ZA-0573
Validated for IHC in human endometrium by the manufacturer.

Anti-CK7 (ZM-0071; ZSGB-BIO)
http://www.zsbio.com/product/ZM-0071
Validated for IHC in human endometrium by the manufacturer.

Human research participants

Policy information about studies involving human research participants

Population characteristics	Patients diagnosed as renal tumor and pRCC were male and female, ages 29-81.
Recruitment	All patients were recruited under an established Institutional Review Board protocol approved by Jinling Hospital and Drum Tower Hospital Affiliated with the Medical School of Nanjing University. Patients were recruited because they were diagnosed as pRCC and undergoing nephrectomy for renal tumor, but no selective recruitment was performed.
Ethics oversight	This study was approved by Jinling Hospital and Drum Tower Hospital Affiliated with the Medical School of Nanjing University under an established Institutional Review Board protocol.We have complied with all relevant ethical regulations with human participants. Informed consent for the human study was provided by all participants.

Note that full information on the approval of the study protocol must also be provided in the manuscript.
